# Chemical gastritis after chronic bromazepam intake: a case report

**DOI:** 10.1186/1471-230X-10-84

**Published:** 2010-07-29

**Authors:** Oliver Kirschberg, Thomas Saers, Bernd Krakamp, Michael Brockmann

**Affiliations:** 1 Department of Internal Medicine II, University of Witten/Herdecke, Campus Cologne-Merheim, Ostmerheimerstrasse 200, D-51109 Cologne, Germany; 2 Department of Pathology, Cologne-Merheim Medical Center (CMMC), Ostmerheimerstrasse 200, D-51109 Cologne, Germany

## Abstract

**Background:**

We describe a rare case of diffuse macroscopic discoloration and chemical gastritis due to chronic bromazepam intake. The chemical composition of pharmaceuticals has to be considered at endoscopy and it is evident that some chemical substances damage the epithelial tissue and lead to clinical symptoms.

**Case Presentation:**

Endoscopy was performed in an 82-year-old patient due to gastroesophageal reflux symptoms and epigastric pain. Gastroscopy showed a hiatal hernia and a scarred duodenal bulb. More striking was the yellow-brownish discoloration of the gastric and the duodenal mucosa. The gastric antrum and the duodenal bulb showed local discoloration that could not be rinsed off. The medical history indicated that bromazepam (6 mg) had been used daily as a sleeping aid in the previous two years. The histopathological findings showed appearances of chemical gastritis. Within the lamina propria and on the epithelial surface there were granules. There was no foreign body reaction to these granules. Corpus mucosa showed a mild chronic gastritis.

**Conclusions:**

If discoloration of the mucosa at endoscopy is seen, a careful drug history must be sought. This is the first case in literature that shows a chemical gastritis after bromazepam intake.

## Background

We describe a rare case of diffuse macroscopic discoloration and chemical gastritis due to chronic bromazepam intake. The chemical composition of pharmaceuticals has to be considered at endoscopy and it is evident that some chemical substances damage the epithelial tissue and lead to clinical symptoms.

## Case Presentation

Endoscopy was performed in an 82-year-old patient due to gastroesophageal reflux symptoms and epigastric pain. Gastroscopy showed hiatal hernia and a scarred duodenal bulb.

**Figure 1:**
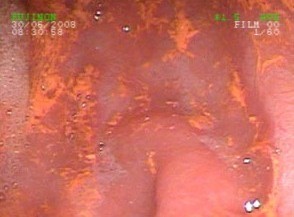
**Endoscopy appearance of the gastric antrum with discoloration after bromazepam intake**.

**Figure 2:**
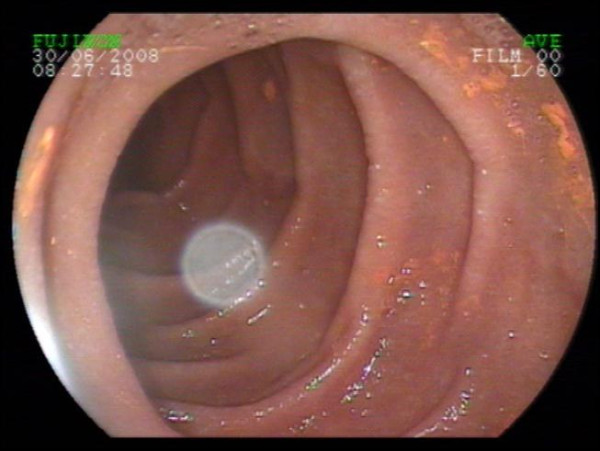
**Discoloration of the duodenal bulb after bromazepam intake**.

More striking was the yellow-brownish discoloration of the gastric and duodenal mucosa. The gastric antrum and the duodenal bulb showed local discoloration that could not be rinsed off (Figures 1 and 2). The medical history indicated that bromazepam (6 mg) had been used daily as a sleeping aid in the previous two years.

**Figure 3:**
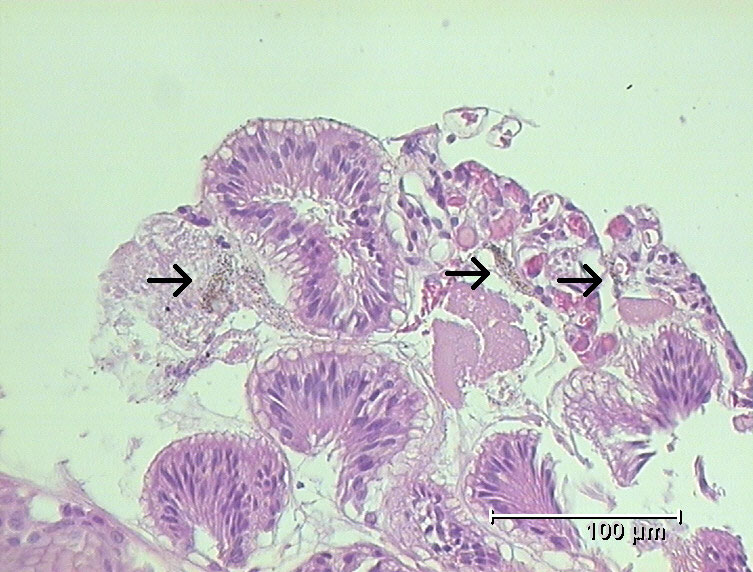
**Histology of gastric antral mucosa**. Arrows show the granules embedded in the mucus and the epithelial surface.

The histopathologic findings of the gastric antrum showed foveolar hyperplasia, smooth muscle proliferation and a scant chronic inflammatory infiltrate with dilated vessels, appearances of chemical gastritis. Within the lamina propria and on the epithelial surface there were granules (Figure 3). There was no foreign body reaction to these granules. Corpus mucosa showed a mild chronic gastritis.

## Conclusions

This is an uncommon example for discoloration of the epithelial surface in the stomach, especially the gastric antrum and the duodenal bulb (Figures 1 and 2). In the literature, only a few examples of discoloration of the gastric mucosa or epithelial surface can be found. In one case Chung et al. [1] found an infectious disease related to a green discoloration in the stomach. Several other reports refer to esophageal injuries after medication intake. Iron tablets [2], doxycycline [3], cyproterone acetate and ethinylestradiol [4] are all described to have led to painful esophagitis. Even a presentation of esophageal cancer-like symptoms after doxycycline intake is described by Tahan et al. [5]. There is little information regarding discoloration of the gastric antrum and duodenal bulb, despite their known propensity to be a hot spot for bleedings, ulcerations, and tumors.

**Figure 4:**
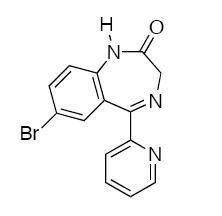
**Chemical composition of bromazepam (C_14_H_10_BrN_3_O)**.

In addition to the macroscopic findings some granules are found microscopically in the mucous that could not be rinsed away (Figure 3). The etiology for this histological finding is not clear. It could be assumed that bromazepam is able to interact with the epithelial surface in an acid milieu. The active pharmaceutical ingredient is bromazepam [(C_14_H_10_BrN_3_O), (Figure 4)] containing a bromo substituent. Elemental bromine is a reactive element. Reactivity is based on its ability to react with certain metals to generate salts. The color of elemental bromine appears in the liquid or gas phase reddish-brown. Yet, any generated bromine in tissue would only be transient, because of its reactive nature. In general medical practice bromine derivatives are used as disinfectants. Bromine is also encountered as a moiety in certain CNS-active drugs (Table 1) [CNS - central nervous system].

**Table 1:** Drugs containing bromine (selection):

Medical drugs:	
pulmonary drugs	bromhexin hydrochloride 8 mg/5 ml

psychotropic drugs or CNS drugs	bromocriptin mesilat 5.735 mg/pill
	bromazepam 6 mg/pill
	bromperidol lactate 5 mg/pill

Iodine, as an example for the same chemical group, is used for coloration of squamous epithelium in the esophagus by absorption in the epithelial cells [6,7]. This discoloration penetrats the mucosa. Thuler et al. describes a chemical esophagitis after using Lugol's solution for detecting esophageal cancer [8]. In contrast to these findings in use of iodine, the bromazepam discoloration refers only to the epithelial surface and the mucus.

This may be a reason for clinical symptoms like epigastric pain. As far as the literature is concerned this is the first case where bromazepam is able to discolor epithelial tissue in specific parts of the stomach.

## Competing interests

The authors declare that they have no competing interests.

## Authors' contributions

OK and BK prepared case report. OK and TS analyzed case report and performed literature research. MB analyzed the specimen and took microscopic photos. All authors read and reviewed the final manuscript.

## Consent

Written informed consent was obtained from the patient for publication of this case report and accompanying images. A copy of the written consent is available for review by the Editor-in-Chief of this journal.

## Pre-publication history

The pre-publication history for this paper can be accessed here:

http://www.biomedcentral.com/1471-230X/10/84/prepub
